# Slip instability behavior of block rock masses on dynamic-static combined loads

**DOI:** 10.1038/s41598-023-36771-4

**Published:** 2023-06-13

**Authors:** Kaixing Wang, Purui Shi, Yishan Pan, V. N. Oparin, Linming Dou

**Affiliations:** 1 School of Mechanics and Engineering, Liaoning Technical University, Liaoning, 123000 Fuxin China; 2 Institute of Mining, Siberian Branch, Russian Academy of Sciences, Novosibirsk, Russia 630091; 3 Key Laboratory of Deep Coal Resource Mining Ministry of Education, China University of Mining Technology, Jiangsu, 221116 Xuzhou China

**Keywords:** Natural hazards, Engineering, Tectonics

## Abstract

A rock mass is a system of various scale blocks embodied into one another. Inter-block layers are usually composed of weaker and fissured rocks. On the action of dynamic-static loads, it can induce slip instability between blocks. In this paper, the slip instability laws of block rock masses are studied. Based on theory and calculation analysis finding that the friction force between rock blocks varies with block vibration and the friction between rock blocks can drop sharply, resulting in slip instability. The critical thrust and occurrence time of block rock masses slip instability are proposed. The factors affecting block slipping instability are analyzed. This study has significance to the rock burst mechanism induced by slip instability of rock masses.

## Introduction

Studying the slip instability of block rock masses under combined static and dynamic loads is of great importance for understanding slip-type rock burst disasters. Lippmann^1^ noted slip and offset under mining in coal seams arise from stiffness contrasts across strata. Zhu et al.^2^ explored mechanism of rock burst in the bottom coal seam of super high seam with overall slippage and instability. Qi et al.^3^ conducted experimental studies on slippage along structures in coal rock and explained rock burst from the perspective of sliding in coal rock. Zhang et al.^4^ studied fault slip-activated roadway rock burst. Sadovsky^5^ proposed that a rock mass exhibits a structural hierarchy of blocks at different scales of self-similarity. Kurlenya^6^ modeled dynamic propagation in block systems with springs and elastic blocks. Jiang et al.^7^ introduced rock friction slip rate and analyzed viscoelasticity and shear effects on ultra-low friction. Aleksandrova et al.^8^ modeling of wave propagation in block media. Cui et al.^9^ studied the effect of lateral stress perturbation on fault friction by experiment. Kurlenya et al.^10,11^ studied the anomalously low friction effect in block media. Li et al.^12^ showed experimental research on energy characteristics of anomalously low friction effect in deep coal and rock mass. Li et al.^13^ used a custom apparatus to simulate in situ stress and study ultra-low friction. Pan et al.^14^ studied ultra-low friction mechanisms based on pendulum wave theory in block rock masses. Wang et al.^15^ studied medium deformation and motion properties of blocks in deep rock masses systems. Wang et al.^16^ indicated that block interface effects depend on block size and the bulk deformation capacity within blocks and at interfaces. Wu et al.^17^ found vertical stress and friction coefficient change in blocks induce slip instability in rock masses. Xu et al.^18^ experimentally studied on friction weakened effect of deep block rock mass and found displacement versus horizontal force is parabolic under vertical impact and static horizontal loading.

Although many researchers have studied slipping laws of block rock masses, the criteria for determination and mechanisms of occurrence remain unclear, requiring further study. This paper studied the laws governing slip instability in block rock masses under combined dynamic and static loading. Based on a mechanical model, criteria for discriminating block slip instability and determining critical thrust are proposed.

## Mechanisms and criteria for slip instability in block rock masses

### Analysis of slip instability of block rock masses under transient disturbance

**Figure 1:**
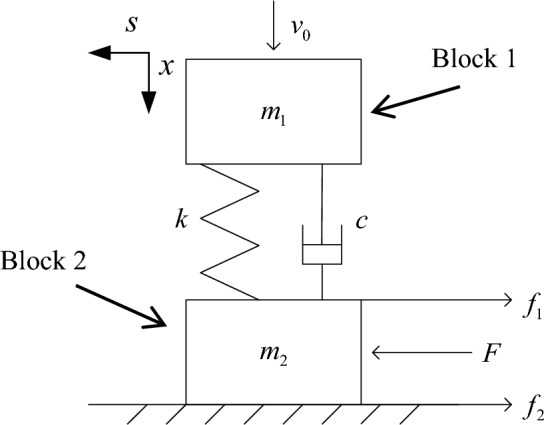
Block rock masses model under transient disturbance.

Basis the paper of Sadovsky^5^, a typical model of block rock masses is depicted in Fig. 1, as proposed in^8,15^ and rock blocks are approximated as rigid bodies interconnected by visco-elastically weaker and fissured interlayers. The masses of blocks are $m_{1}$ and $m_{2}$, respectively. $k$ and $c$ are the stiffness and viscosity of the weak medium between blocks. $v_{0}$ is the initial velocity of block 1 under transient disturbance. $F$ is the static load on block 2 in the horizontal direction. $f_{1}$ and $f_{2}$ are the frictional forces of block 2.

The kinetic equation for block $m_{2}$ in the horizontal direction is given as Eq. (1).

1 $$F - f = m_{2} a$$

where $f$ is the frictional force between block 2 and the contact surface, expressed as Eq. (2).

2 $$f = f_{1} + f_{2}$$

where $f_{{1}}$ represents the friction between block 1 and block 2, as Eq. (3), and $f_{{2}}$ represents the friction between block 2 and the fixed interface, as Eq. (4).

3 $$f_{1} = [m_{1} g + F_{k} ] \cdot \mu$$

4 $$f_{2} = [(m_{1} + m_{2} )g + F_{k} ] \cdot \mu$$

where $F_{k} = kx$ and $x$ is the relative displacement between block 1 and block 2 in the vertical direction and $\mu$ is friction coefficient. Therefore, the slip acceleration of block 2 in horizontal direction is expressed as Eq. (5).

5 $$a = \frac{{F - \left[ {2m_{1} g + m_{2} g + 2kx} \right] \cdot \mu }}{{m_{2} }}$$

The kinetic energy and slip displacement of block 2, respectively as Eqs. (6) and (7).

6 $$E_{hk}^{{}} = \frac{1}{2}m_{2} \left( {\int_{0}^{t} {adt} } \right)^{2}$$

7 $$s = \int_{0}^{t} {\int_{0}^{t} a d^{2} t}$$

For block 2 to slip, inequality (8) must be satisfied.

8 $$m_{2} a = F - f > 0$$

From inequality (8), the critical horizontal thrust $F_{c}$ of block 2 can be determined, as Eq. (9).

9 $$F_{c} = \min [f] = \min [(2m_{1} g + m_{2} g + 2kx)\mu ]$$

$F_{c}$ represents the minimum thrust required for block 2 to slip. As evidenced in Eq. (9), $F_{c}$ is a function related to the displacement $x$ of block 1. The kinetic equation for block 1, under transient perturbations, as Eq. (10).

10 $$\left\{ {\begin{array}{*{20}l} {m_{1} \ddot{x}(t) + c\dot{x}(t) + kx(t) = 0} \hfill \\ {x(0) = 0} \hfill \\ {\dot{x}(0) = v_{{0}} } \hfill \\ \end{array} } \right.$$

From^19^, the solution of Eq. (10) is obtained as Eq. (11).

11 $$x(t) = \frac{{v_{0} }}{{\omega_{d} }}\sin (\omega_{d} t)e^{{ - \xi \omega_{n} t}}$$

where $\xi = \frac{c}{{c_{c} }}$, $c_{c} = 2\sqrt {km_{{1}} }$; $\omega_{n} = \sqrt {\frac{k}{{m_{1} }}}$; $\omega_{d} = \omega_{n} \sqrt {1 - \xi^{2} }$. Substituting Eq. (11) into Eq. (9) yields the critical horizontal thrust, $F_{c}$, as Eq. (12). The time $t$ of critical horizontal thrust occurrence, as Eq. (13).

12 $$F_{c} = \left( {2m_{1} g + m_{2} g - 2k\frac{{v_{0} }}{{\omega_{d} }}e^{{ - \xi \omega_{n} \frac{{{3}\pi }}{{2\omega_{d} }}}} } \right)\mu$$

13 $$t = \frac{{{3}\pi }}{{2\omega_{d} }}$$

The resultant force of block 1 in the vertical direction, as Eq. (14).

14 $$F_{r}^{(t)} = m_{1} \ddot{x}$$

The kinetic energy, $E_{k}$, of block 1 and the potential energy, $E_{p}$, between blocks weak medium, are as Eq. (15).

15 $$E_{k} = \frac{1}{2}m_{1} \dot{x}_{{}}^{2} ,E_{p} = \frac{1}{2}kx_{{}}^{2}$$

### Analysis of slip instability of block rock masses under steady state disturbance

**Figure 2:**
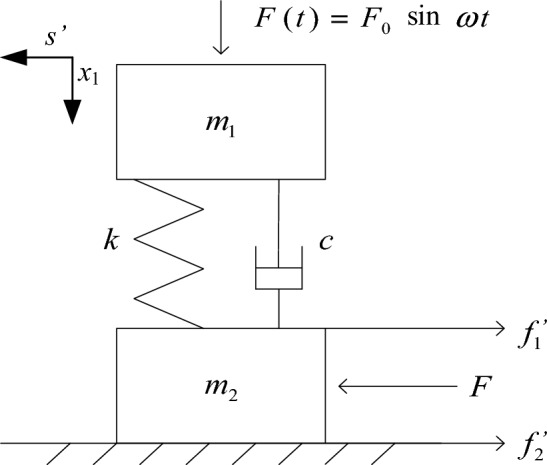
Block rock masses model under steady state disturbance.

Block rock masses slip instability under steady state disturbance is analyzed using the model in Fig. 2. The difference between Figs. 1 and 2 is only in the disturbance, here $F(t) = F_{0} \sin \omega t$.

In Fig. 2, the friction of block 2 can be expressed as Eqs. (16)–(18).

16 $$f^{\prime} = f_{1}^{\prime } + f_{2}^{\prime }$$

17 $$f^{\prime}_{1} = [m_{1} g + F^{\prime}_{k} + F(t)] \cdot \mu$$

18 $$f^{\prime}_{2} = [(m_{1} + m_{2} )g + F^{\prime}_{k} + F(t)] \cdot \mu$$

where $F^{\prime}_{k} = kx_{1}$. The slip acceleration of block 2 in horizontal direction is expressed as Eq. (19).

19 $$a^{\prime} = \frac{{F - \left[ {2m_{1} g + m_{2} g + 2kx_{1} + 2F(t)} \right] \cdot \mu }}{{m_{2} }}$$

The kinetic energy and slip displacement of block 2, respectively as Eqs. (20) and (21).

20 $$E^{\prime}_{hk} = \frac{1}{2}m_{2} \left( {\int_{0}^{t} {a^{\prime}dt} } \right)^{2}$$

21 $$s^{\prime} = \int_{0}^{t} {\int_{0}^{t} {a^{\prime}} d^{2} t}$$

The kinetic equation of block 1 under steady state disturbance can be expressed as Eq. (22).

22 $$m_{{1}} \ddot{x}_{{1}} + c\dot{x}_{{1}} + kx_{{1}} = F_{0} \sin \omega t$$

From^19^, the solution of Eq. (22), as Eq. (23).

23 $$x_{1} = X_{0} \sin (\omega t - \varphi )$$

where, $$\left\{ \begin{gathered} X_{0} = \frac{{F_{0} }}{{k\sqrt {(1 - \overline{\omega }^{2} )^{2} + (2\xi \overline{\omega })^{2} } }} \hfill \\ \varphi = \arctan \frac{{2\xi \overline{\omega }}}{{1 - \overline{\omega }^{2} }} \hfill \\ \end{gathered} \right.$$ and $\overline{\omega } = \frac{\omega }{{\omega_{n} }}$. The critical horizontal thrust under steady state disturbance, $F^{\prime}_{c}$, as Eq. (24).

24 $$F^{\prime}_{c} = \min [f^{\prime}] = \min [(2m_{1} g + m_{2} g + 2F(t) + 2kx_{1} )\mu ]$$

By substituting Eq. (23) into (24), $F^{\prime}_{c}$ can be expressed as Eq. (25).

25 $$F^{\prime}_{c} = (2m_{1} g + m_{2} g - \sqrt {L_{1}^{2} + N_{1}^{2} } ) \cdot \mu$$

where, $L_{1} = 2F_{0} + 2kX_{0} \cos \varphi$, $N_{1} = 2kX_{0} \sin \varphi$, and the time $t^{\prime}$ of critical horizontal thrust $F^{\prime}_{c}$ occurrence, satisfies Eq. (26).

26 $$t^{\prime} = \frac{\theta + 3\pi /2}{\omega }$$

where $\theta = \arctan \frac{{N_{1} }}{{L_{1} }}$. The resultant force of block 1 in the vertical direction, as Eq. (27).

27 $$F_{r}^{(s)} = m_{1} \ddot{x}_{1}$$

The kinetic energy, $E^{\prime}_{k}$, of block 1 and the potential energy, $E^{\prime}_{p}$, between blocks weak medium, are as Eq. (28).

28 $$E^{\prime}_{k} = \frac{1}{2}m_{1} (\dot{x}_{1} )^{2} ,E^{\prime}_{p} = \frac{1}{2}k(x_{1} )^{2}$$

## Calculation and analysis of slip instability in block rock masses

### Slip instability in block rock masses under transient disturbance

**Figure 3:**
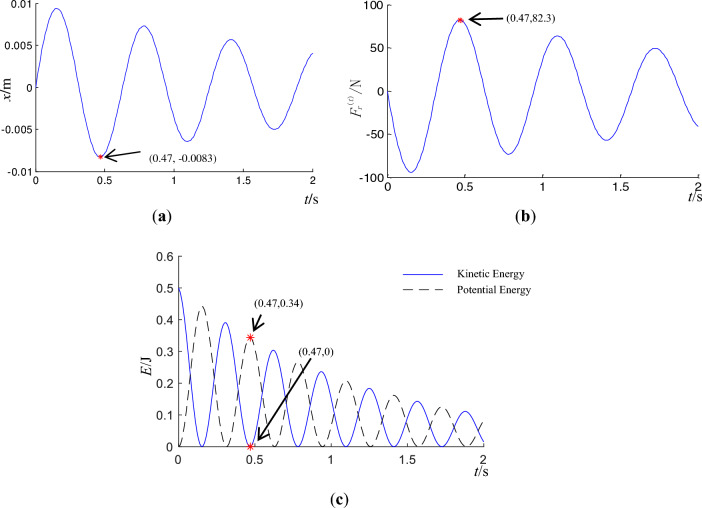
Dynamic response of block 1 in disturbance direction. (**a**) displacement of block $m_{1}$. (**b**) resultant force of block 1. (**c**) kinetic energy of block 1 and potential energy of weak medium between blocks.

Based on the mechanical model of block rock masses shown in Fig. 1 and the calculation parameters from Refs.^14,20^, the original calculation parameters selected in this paper are $v_{0} = 0.1$ m/s, $m_{1} = m_{2} = 100$ kg, $k = 10000$ kg/s^2^, $c = 80$ kg/s, and $\mu = 0.4$. The displacement $x$, resultant force $F_{r}^{(t)}$, kinetic energy $E_{k}$ of block 1, and potential energy of the weak medium between the blocks $E_{p}$ are shown in Fig. 3.

**Figure 4:**
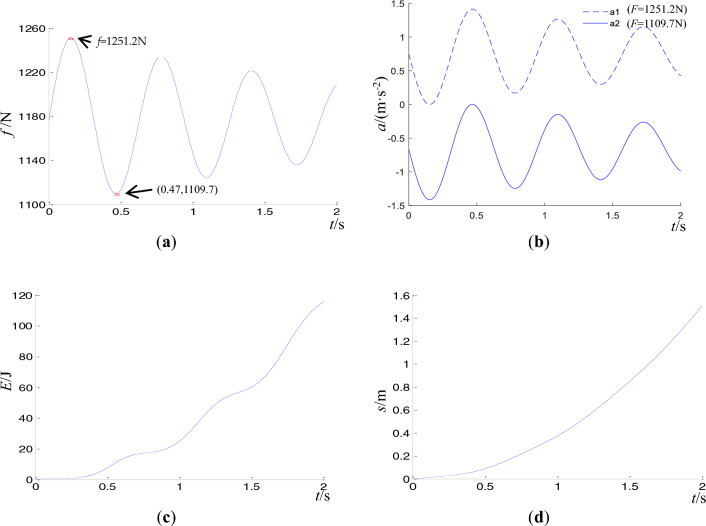
Dynamic response of the sliding block 2 in horizontal direction. (**a**) friction of block 2. (**b**) acceleration of block 2. (**c**) kinetic energy of block 2 on horizontal thrust *F* = 1251.2 N. (**d**) displacement of block $m_{2}$ on horizontal thrust *F* = 1251.2 N.

In Fig. 3a, at *t* = 0.47 s, the displacement, *x*, reaches a minimum of − 0.0083 m, indicating the maximum separation of the two blocks. In Fig. 3b, when *t* = 0.47 s, the resultant force of block 1 in the vertical direction reaches a maximum of 82.3 N. In Fig. 3c, kinetic and potential energy are converted into each other during the dynamics process. At *t* = 0.47 s, the potential energy reaches a periodic extreme value of 0.34 J, while the kinetic energy approaches zero. The friction force $f$, acceleration $a$, slip displacement $s$, and kinetic energy of block 2 in horizontal direction $E_{k}^{(h)}$ are depicted in Fig. 4.

In Fig. 4a, at *t* = 0.47 s, the friction force of block 2 reaches a minimum of 1109.7 N, which agrees with Eqs. (12) and (13), while the maximum friction force is 1251.2 N. In Fig. 4b, under the action of the horizontal thrust *F* = 1109.7 N, the acceleration of block 2 is less than zero except at *t* = 0.47 s. This means that block slip occurs only at *t* = 0.47 s. But under the action of the horizontal thrust *F* = 1251.2 N, the acceleration is always greater than zero and reaches a maximum at *t* = 0.47 s. Therefore, block sliding is related to horizontal thrust. In Fig. 4c and d, the kinetic energy of block 2 sliding increases stepwise with time, which is due to the periodic tensile and compressive action between the blocks, and the displacement shows exponential growth.

### Slip instability in block rock masses under steady-state disturbance

**Figure 5:**
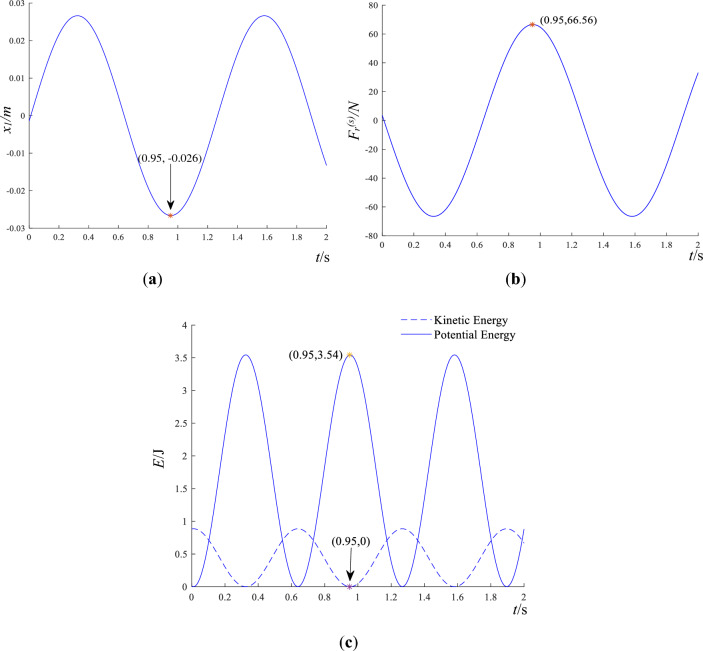
Dynamic response of block 1 on steady state disturbance. (**a**) displacement of block 1. (**b**) resultant force of block 1. (**c**) kinetic energy of block 1 and potential energy of weak medium between blocks.

Based on the mechanical model of block rock masses depicted in Fig. 2, and the calculation parameters $m_{1}$,$m_{2}$, $k$, $c$, $\mu$ are the same as Section “Slip instability in block rock masses under transient disturbance”, and $F(t) = F_{0} \sin (\omega t) = 200\sin (5t)$. The displacement $x_{1}$, resultant force $F_{r}^{(s)}$, kinetic energy $E^{\prime}_{k}$, of block 1 and the potential energy of the weak medium between blocks $E^{\prime}_{p}$ are shown in Fig. 5.

**Figure 6:**
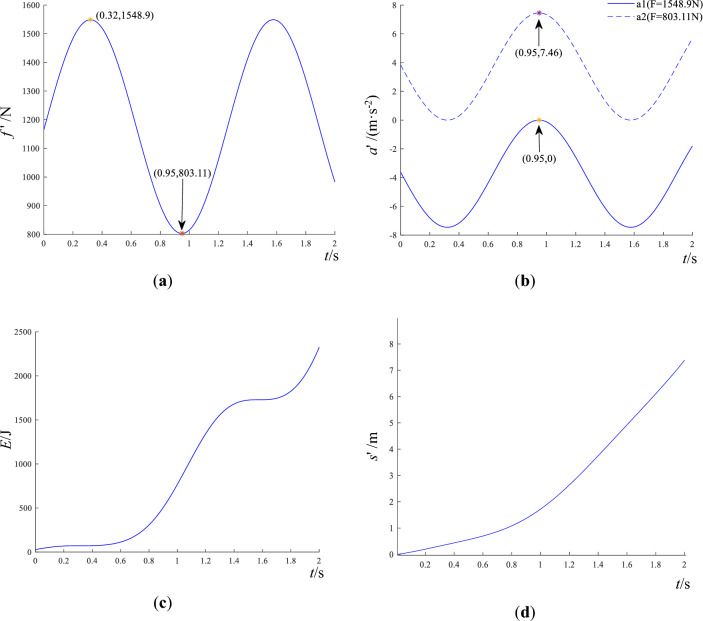
Dynamic response of the sliding block 2 on steady state disturbance. (**a**) friction of sliding block 2. (**b**) acceleration of sliding block 2. (**c**) kinetic energy of sliding block 2 on horizontal thrust *F* = 1548.9 N. (**d**) displacement of sliding block 2 on horizontal thrust *F* = 1548.9 N.

In Fig. 5a, the displacement of block 1 reaches a minimum at *t* = 0.95 s, indicating the maximal separation between the two blocks. In Fig. 5b, at *t* = 0.95 s, the resultant force of block 1 in the vertical direction reaches a maximum. In Fig. 5c, the kinetic energy and potential energy transform into each other during the dynamics process. At *t* = 0.95 s, potential energy reaches a periodic extreme value while kinetic energy is close to zero. Because the steady-state disturbance $F(t) = 200\sin (5t)$ results in the deformation of weak medium, and the potential energy in Fig. 5c is higher than the kinetic energy. The friction force $f^{\prime}$, acceleration $a^{\prime}$, slip displacement $s^{\prime}$, and kinetic energy $E^{\prime}_{hk}$ of block 2 in the horizontal direction are depicted in Fig. 6.

**Figure 7:**
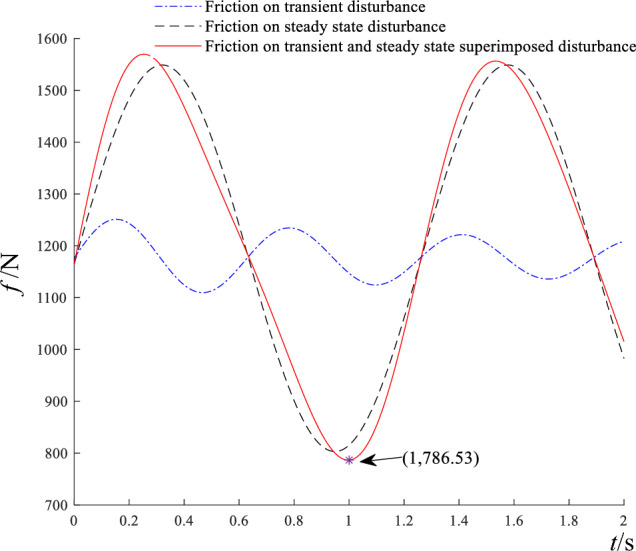
The friction force of block 2 on external disturbances.

In Fig. 6a, the friction force reaches a minimum of $F^{\prime}_{c} = 803.11$ N at $t^{\prime} = 0.95$ s, which agrees with Eqs. (25) and (26). The maximum friction is $F^{\prime}_{m} = 1548.9$ N. In Fig. 6b, under the action of horizontal thrust $F^{\prime}_{c} = 803.11$ N, the acceleration is negative except at $t^{\prime} = 0.95$ s. It indicates that block slip occurs only at $t^{\prime} = 0.95$ s. But under the load of horizontal thrust $F^{\prime}_{m} = 1548.9$ N, the acceleration is always positive, and acceleration peaks at $t^{\prime} = 0.95$ s, indicating that slip instability is most likely at this instant. In Fig. 6c and d, the kinetic energy of slider 2 increases stepwise with time, due to the periodic tensile and compressive action between blocks and the exponentially growing displacement. The friction forces acting on block 2 in different kinds of disturbances are demonstrated in Fig. 7.

In Fig. 7, when *t* = 1 s, the friction force attains a minimum of 786.53 N on the combined effects of transient and steady-state disturbances. From Fig. 6a, the friction force descends to a minimum of 803.11 N at *t* = 0.95 s, on steady-state disturbance. From Fig. 4a, the friction force drops to a minimum of 1109.7 N at *t* = 0.47 s, on transient disturbance. Consequently, the combined effect of the two disturbances modifies the friction force of block slipping.

## Discussion

In summary, under the combined action of dynamic and static loads, this study investigates the slip instability behavior of underlying block rock in response to transient and steady-state disturbances, respectively. We found that during the non-coordinated dynamic response process of the block rock masses, periodic tension and compression changes exist between the blocks, and the corresponding frictional force between the blocks also changes with time. There is a physical process of a sudden drop in frictional force between the blocks, resulting in block slip instability. The block slip is also related to the rock interface friction coefficient; therefore, the effect between friction coefficient and block slip velocity can be further studied in the future.

## Conclusions

1. The mechanical mechanism of friction forces sudden drop between rock blocks within block rock masses is analyzed. The fluctuation of friction force between blocks is related to the non-coordinated dynamic response of the block rock masses.
2. The critical thrust and occurrence time of block sliding under transient and steady-state dynamic and static loads are given by theory, which is of great significance for predicting the sliding instability of block rock masses.

## Data Availability

The datasets used and/or analysed during the current study available from the corresponding author on reasonable request.
